# Performance of Artificial Intelligence-Based Algorithms to Predict Prolonged Length of Stay after Lumbar Decompression Surgery

**DOI:** 10.3390/jcm11144050

**Published:** 2022-07-13

**Authors:** Babak Saravi, Alisia Zink, Sara Ülkümen, Sebastien Couillard-Despres, Frank Hassel, Gernot Lang

**Affiliations:** 1 Department of Orthopedics and Trauma Surgery, Medical Center—University of Freiburg, Faculty of Medicine, University of Freiburg, 79108 Freiburg, Germany; gernotmichaellang@gmail.com; 2 Department of Spine Surgery, Loretto Hospital, 79108 Freiburg, Germany; alisia.zink@gmail.com (A.Z.); sara.uelkuemen@jupiter.uni-freiburg.de (S.Ü.); frank.hassel@rkk-klinikum.de (F.H.); 3 Institute of Experimental Neuroregeneration, Spinal Cord Injury and Tissue Regeneration Center Salzburg (SCI-TReCS), Paracelsus Medical University, 5020 Salzburg, Austria; s.couillard-despres@pmu.ac.at; 4 Austrian Cluster for Tissue Regeneration, 1200 Vienna, Austria

**Keywords:** length of stay, spine surgery, decompression, spinal stenosis, machine learning, deep learning, artificial intelligence, prediction

## Abstract

Background: Decompression of the lumbar spine is one of the most common procedures performed in spine surgery. Hospital length of stay (LOS) is a clinically relevant metric used to assess surgical success, patient outcomes, and socioeconomic impact. This study aimed to investigate a variety of machine learning and deep learning algorithms to reliably predict whether a patient undergoing decompression of lumbar spinal stenosis will experience a prolonged LOS. Methods: Patients undergoing treatment for lumbar spinal stenosis with microsurgical and full-endoscopic decompression were selected within this retrospective monocentric cohort study. Prolonged LOS was defined as an LOS greater than or equal to the 75th percentile of the cohort (normal versus prolonged stay; binary classification task). Unsupervised learning with K-means clustering was used to find clusters in the data. Hospital stay classes were predicted with logistic regression, RandomForest classifier, stochastic gradient descent (SGD) classifier, K-nearest neighbors, Decision Tree classifier, Gaussian Naive Bayes (GaussianNB), support vector machines (SVM), a custom-made convolutional neural network (CNN), multilayer perceptron artificial neural network (MLP), and radial basis function neural network (RBNN) in Python. Prediction accuracy and area under the curve (AUC) were calculated. Feature importance analysis was utilized to find the most important predictors. Further, we developed a decision tree based on the Chi-square automatic interaction detection (CHAID) algorithm to investigate cut-offs of predictors for clinical decision-making. Results: 236 patients and 14 feature variables were included. K-means clustering separated data into two clusters distinguishing the data into two patient risk characteristic groups. The algorithms reached AUCs between 67.5% and 87.3% for the classification of LOS classes. Feature importance analysis of deep learning algorithms indicated that operation time was the most important feature in predicting LOS. A decision tree based on CHAID could predict 84.7% of the cases. Conclusions: Machine learning and deep learning algorithms can predict whether patients will experience an increased LOS following lumbar decompression surgery. Therefore, medical resources can be more appropriately allocated to patients who are at risk of prolonged LOS.

## 1. Introduction

In patients over 65, lumbar spinal stenosis (LSS) is the most commonly occurring reason for spinal surgery, and decompression of the lumbar spine is among the most common surgical procedures [1]. In theory, surgical decompression should relieve discomfort and improve function. In addition to laminectomy and laminotomy, fusion procedures may also be used as decompression methods. The purpose of spinal surgery is to reduce the pressure on the nerves afflicted by spinal stenosis by improving the cross-sectional area of the spinal canal [2]. Nearly a third of the general population suffers from lower back pain. Approximately 80 to 100 billion USD are spent annually on treating these patients [3,4]. The annual incidence of LSS surgery in the United States is approximately 1,2 per 1000; however, the prevalence of LSS in adults is expected to rise 59% by the year 2025 [5,6]. In terms of hospital expenses alone, the total cost of surgical intervention amounted to 1.65 billion USD [4]. An estimated 306 million USD have been spent on lumbar spine surgeries in the United States in the last few years among patients aged 65 and older [4,7,8]. In particular, by 2050, the elderly population is projected to increase from 43.1 million people to 83.7 million people, which will result in a significant increase in healthcare expenditures on degenerative spine diseases. An average of 8–10% of all patients who undergo spinal decompression need to undergo the procedure again, resulting in higher hospital costs [9,10]. Furthermore, these surgical procedures carry a 3.1% risk of cardiac problems or stroke, as well as a 0.4% risk of death within one month [4].

An important factor to consider when evaluating socioeconomic costs, surgical success, and patient outcomes is the length of stay (LOS). LOS is a measure that is frequently used to reduce healthcare costs, especially in light of escalating healthcare expenditures. When patients are undergoing spinal surgery, the length of stay variable is critical from the patient’s point of view and is a major factor in determining health care expenditures. It is estimated that each additional day in the hospital costs approximately 1000 USD [11], and inpatient hospital expenditures (excluding instrumentation and surgical procedures) strongly correlate with the length of stay [12]. It is estimated that hospitalization costs for spine surgery increased nearly threefold between 1992 and 2003 [13,14]. Hospitalization costs are significant and should not be underestimated. In order to define value-based care for spinal illnesses, it is crucial to determine the precise length of stay. Ensuring that patients are not kept in the hospital for longer than necessary is an important goal. An analysis conducted by Boylan and colleagues on the costs associated with adolescents’ scoliosis surgery found that each additional day of hospitalization costs in excess of 1100 USD in insurance expenditures and in excess of 5200 USD in terms of hospital charges [15]. Additionally, long-term hospital patients can incur an additional 19,000 USD in costs in comparison to those with a shorter hospital stay [16]. For other spinal fusion surgeries, similar results have been observed, indicating the need for further research either to reduce LOS significantly or to prevent unnecessary extensions [17].

Predictive analysis and machine learning have emerged as valuable tools for predicting patient outcomes based on pertinent feature characteristics variables [18]. Developing patient-centered outcome prediction models, including those for patient-related outcome measures and length of stay, can contribute to improving society’s utilization of healthcare resources [19,20]. In doing so, policymakers and clinicians could compare treatments across disciplines to determine how best to allocate budget resources among different approaches.

Many studies have already shown that certain characteristics are associated with a longer hospital stay following spinal surgery. A longer length of stay (LOS) was associated with an increase in operating time during adult spinal deformity surgery, according to Phan et al. [15]. The authors of a multicenter study found that age, the number of levels fused, infection, and comorbidities are risk factors for a higher death rate [16]. Despite their ability to identify individuals likely to have a longer LOS, risk indicators cannot predict whether a patient will have a longer LOS. The use of machine learning and deep learning algorithms can enhance the knowledge provided by these studies and predict whether a particular patient will require a normal length of hospital stay or an extended hospital stay based on a wide range of clinicopathological characteristics. In particular, these algorithms can be integrated into the hospital’s software environment, resulting in continuous monitoring of at-risk patients and the achievement of precision medicine goals.

Considering the socioeconomic impact of prolonged LOS, the present study sought to investigate various artificial intelligence-based algorithms that might be able to predict whether lumbar decompression surgery patients will experience a short or long LOS. Further, we aim to find the most relevant feature variables which are important to solve this classification task.

## 2. Materials and Methods

### 2.1. Study Design

We performed a retrospective cohort study including consecutive patients treated with microsurgical decompression or full-endoscopic interlaminar decompression of lumbar spinal stenosis between 2016 and 2021 at the Department of Spine Surgery, Loretto-Hospital Freiburg in Germany, an affiliated hospital of the University Medical Center Freiburg, Germany. This retrospective observational study was approved by the local Ethics Committee Freiburg, Germany [Number: 116/200]. Written informed consent to participate in observational studies was obtained from each patient.

The main inclusion criterion involved patients with lumbar spinal stenosis treated with either microsurgical or full-endoscopic decompression in the aforementioned period. The iLESSYS^®^ system (Joimax GmbH, Karlsruhe, Germany) was utilized for the endoscopic group. After collecting all data from patients fulfilling our inclusion criteria, we applied our exclusion criteria for filtering the initial dataset. Exclusion criteria included: <18-years-old patients, patients with tumors of the spine, patients having spinal fusion, and patients who have declined the usage of their data for research purposes.

### 2.2. Data Handling and Statistical Analysis

Patients were collected from the in-house patient information system and extracted into a predefined datasheet. Data was pseudonymized utilizing a code generated with the “encode” command in Stata Statistical Software Release 15 (StataCorp. 2011, College Station, TX, USA). Variables were included in the study extraction form according to our previous study [20] and a literature search to only consider previously identified significant variables for hospital length of stay. The surgery-related and clinical factor variable group included surgical technique (microsurgical versus full-endoscopic decompression), number of targeted levels, operation time (OT), hospital length of stay (LOS), the American Society of Anesthesiologists (ASA) physical status classification, and perioperative and postoperative complications. Patient data were screened for the following complications: residual sensorimotor deficits or new-onset sensorimotor deficits, hematomas requiring revision, persisting stenosis requiring revision, postoperative instability, and fracture. This group also contained the demographic data for descriptive statistics (sex, age, BMI), alcohol and nicotine use data, and German insurance type (private or public insurance). The laboratory variable group included preoperative C-reactive protein (CRP) levels.

The target classes were classified as prolonged when hospital length of stay was ≥75% percentile and normal when <75% percentile (binary classification task) [21,22]. Unsupervised learning with K-means clustering was used to evaluate whether data can be clustered according to the features in the dataset. Auto clustering was used according to Schwarz’s Bayesian Criterion (BIC). Comparison of cluster variables was made with the Mann–Whitney U test or Chi-square test where applicable. Further, a decision tree with the Chi-square automatic interaction detection algorithm was applied. CHAID is a statistical tool to find the difference between child and parent nodes. The difference between observed and expected counts of the target variable for each node and the squared sum of these standardized differences will give the Chi-square value. CHAID algorithm was applied with cross-validation (*n* = 5), maximum tree depth of 3, minimum cases in parent node 10, and minimum cases in child node of 5, resulting in *n* = 19 nodes, *n* = 11 terminal nodes, and a depth of 3. Multiple imputations were applied utilizing linear regression for scale variables and logistic regression for categorical variables to impute missing values for the CHAID algorithm. Supervised machine learning and deep learning techniques were applied to predict the target LOS classes. The following algorithms were used: logistic regression, RandomForest classifier, stochastic gradient descent (SGD) classifier, K-nearest neighbors, Decision Tree classifier, Gaussian Naive Bayes (GaussianNB), support vector machines (SVM), a custom-made convolutional neural network (CNN), multilayer perceptron artificial neural network (MLP), and radial basis function neural network (RBNN). The hardware and software environment specifications were as follows:

- CPU: AMD Ryzen 9 5950X 16-Core Processor (Santa Clara, CA, USA)
- RAM: 64 GB
- GPU: NVIDIA Geforce RTX 3090 (Santa Clara, CA, USA)
- Python version: 3.10.4 (64-bit) (Wilmington, DE, USA)
- OS: Windows 10 (Redmond, WA, USA)

Statistical analyses were conducted in Python and SPSS v26 (IBM, Armonk, NY, USA).

## 3. Results

**Figure 1:**
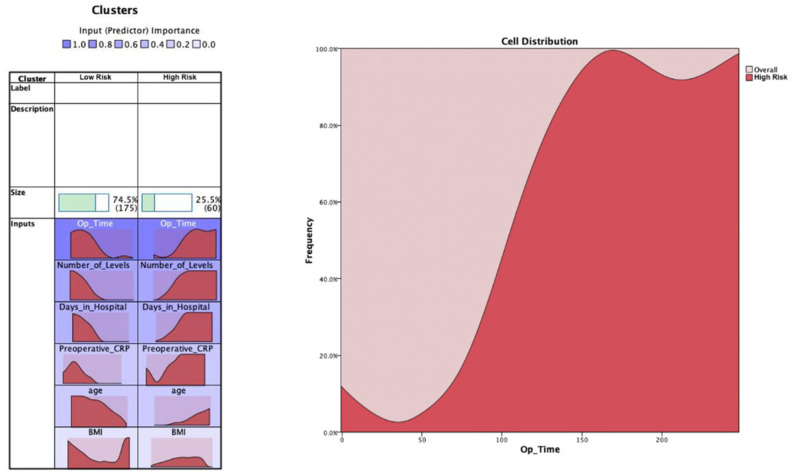
K-means cluster analysis for the raw hospital length of stay target variable (continuous scale). The most important feature to classify the data was the operation time, followed by the number of levels, LOS, preoperative CRP, age, and BMI. The distribution charts show the distribution of the features for both clusters. An example feature (operation time) is shown to help interpretation. The selected feature shows that the distribution of operation time is right-shifted for the high risk cluster, whereas it is left-shifted for the low risk cluster.

We first performed unsupervised learning via K-means cluster analysis to cluster the dataset into two classes and evaluate which features are important for clustering. The first cluster analysis was done with the hospital length of stay (LOS) as raw data type (continuous scale). The cluster analysis resulted in two clusters (74.5% in the high risk group and 25.5% in the low risk group) (Figure 1). The data distribution showed that all cluster variables (BMI, number of levels, age, preoperative CRP, and LOS) were significantly higher for the high risk cluster than low risk cluster (*p* < 0.0001 for all comparisons) (Table 1). Overall, it was possible to find two clusters indicating patients’ risk levels (i.e., the high risk group showed higher BMI, number of targeted levels, age, LOS, and preoperative CRP).

**Figure 2:**
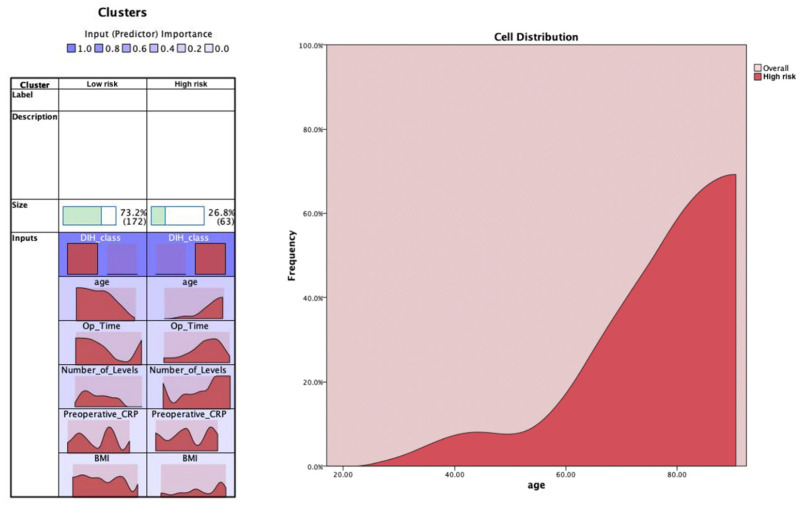
K-means cluster analysis for the binary hospital length of stay class (normal versus prolonged). The most important feature to classify the data was the hospital length of stay class (DIH_class), followed by age, operation time, number of levels, preoperative CRP, and BMI. The distribution charts show the distribution of the features for both clusters. An example feature (age) is shown to help interpretation. The selected feature shows that age distribution is right-shifted for the high risk cluster (including the prolonged hospital stay class), whereas it is left-shifted for the low risk cluster.

The second cluster analysis was done with the hospital length of stay (LOS) as a binary class type utilizing the 75% percentile as the cut-off point. The cluster analysis resulted in two clusters (26.8% in cluster 1 and 73.2% in cluster 2) (Figure 2). The data distribution showed that all cluster variables (BMI, number of levels, age, preoperative CRP) were significantly higher for cluster number 1 than cluster number 2 (*p* < 0.0001 for all comparisons) (Table 2). Further, the distribution of LOS class was significantly different between clusters 1 and 2, with all prolonged cases being in cluster 1 and normal cases being in cluster 2 (*p* < 0.0001).

**Figure 3:**
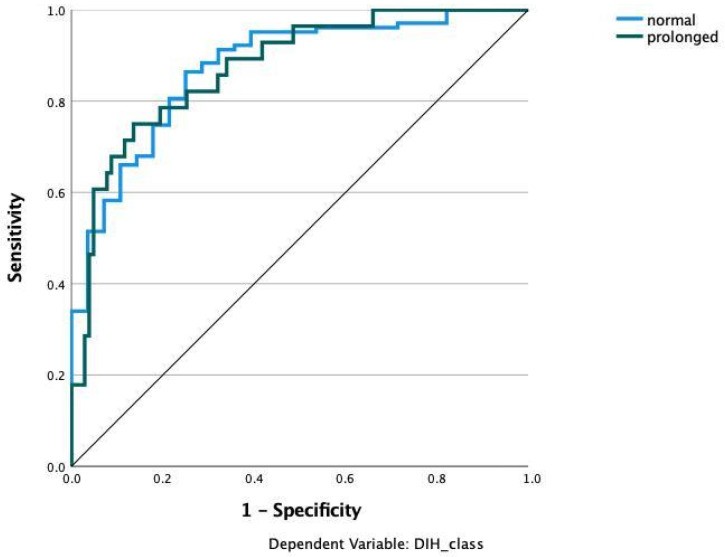
Prediction of hospital stay with multilayer perceptron (MLP). Input layer: feature variables (22 units). Hidden Layer: 2 units, activation function: hyperbolic tangent. Output layer: dependent variable Hospital stay (2 units), activation function: softmax, error function cross-entropy. The number of units in the hidden layer was determined by the testing data criterion: the best number of hidden units is the one that yields the smallest error in the testing dataset. Train/Test/Validation split: 80/10/10. Percent incorrect predictions on training set: 14.5%; percent incorrect predictions on testing set: 14.3%; percent incorrect predictions on holdout set: 10.0%; 0 refers to the prediction of the normal class; 1 refers to the prediction of the prolonged LOS class. AUC: 0.873.

**Figure 4:**
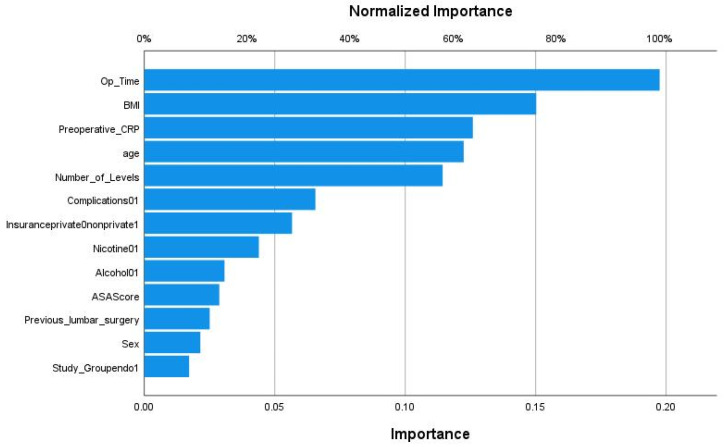
Feature importance analysis for predicting the hospital length of stay classes in the multilayer perceptron model.

We applied various machine learning and deep learning algorithms to predict the LOS classes in the next step. The AUC for the multilayer perceptron model reached the highest AUC with 0.873 (Figure 3). The feature importance analysis revealed that operation time was the most important feature for the classification task, followed by BMI, preoperative CRP, and age (Figure 4). 0: normal LOS; 1: prolonged LOS.

**Figure 5:**
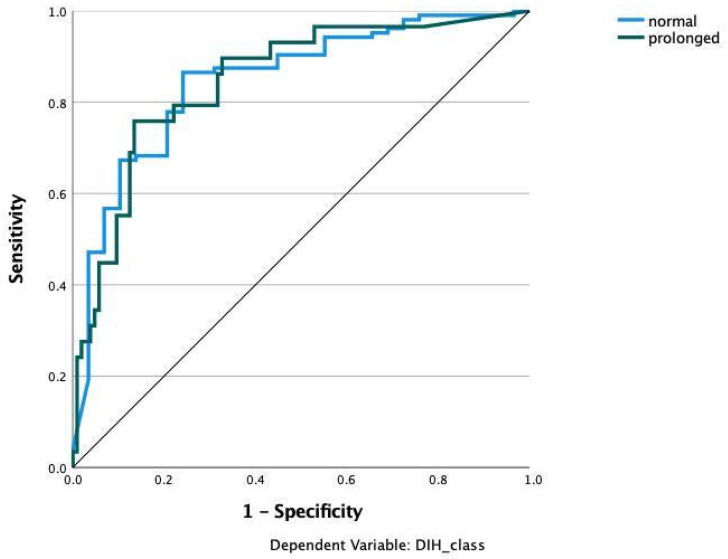
Prediction of hospital stay with radial basis function neural network (RBNN). Input layer: feature variables (22 units). Hidden Layer: 5 units, activation function softmax. Output layer: dependent variable Hospital stay (2 units), activation function: identity, error function sum of squares. The number of units in the hidden layer was determined by the testing data criterion: the best number of hidden units is the one that yields the smallest error in the testing dataset. Train/Test/Validation split: 80/10/10. Percentage incorrect predictions on the training set 19.0%; percent incorrect predictions on testing set: 11.8%; percent incorrect predictions on holdout set: 16.7%. AUC: 0.847.

**Figure 6:**
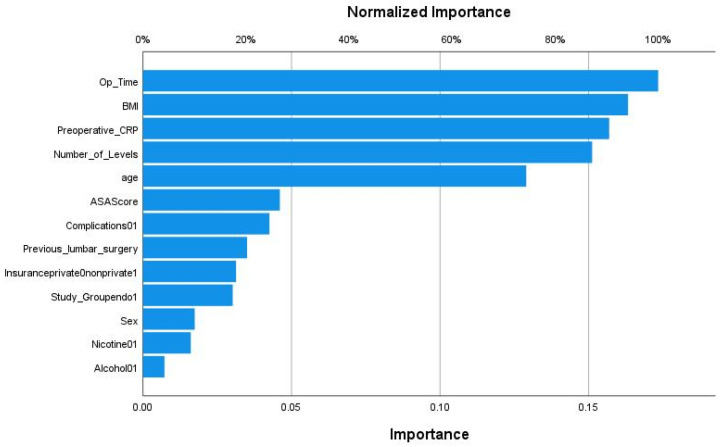
Feature importance analysis for predicting the hospital length of stay classes in the radial basis function neural network model (RBNN).

The AUC for the radial basis function neural network model (RBNN) reached an AUC of 0.847 (Figure 5). The MLP model’s feature importance analysis revealed that operation time was the most important feature for the classification task, followed by BMI and preoperative CRP. However, the number of levels reached higher importance than age with this model age (Figure 6).

The accuracies and AUC of the machine learning algorithms and the custom-made convolutional neural network (CNN) is shown in Table 3. The logistic regression reached the highest accuracy, whereas the highest AUC was reached for the custom-made CNN (Table 3).

**Figure 7:**
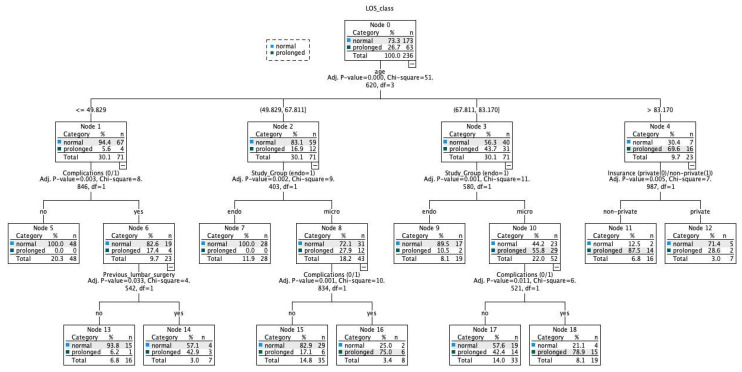
Decision tree with CHAID growing method. Five-fold cross-validation. Percent correct predictions with this algorithm: 84.7%.

Finally, we developed a decision tree utilizing the CHAID growing method for simple interpretation in clinics (Figure 7). The algorithm could detect 84.7% of the cases correctly. How to read the CHAID decision tree: for example, an age ≤ 49.829 resulted in 94.4% of normal and only 5.6% of prolonged LOS cases. An absence of complication after this node further results in all cases having normal LOS. In addition, an age between 49.829–67.811 results in 83.1% of cases having normal LOS. Of these cases who underwent endoscopic decompression, all patients had normal LOS. In contrast, an age of >83.170 results in most cases having prolonged LOS (69.6%). Most of these cases had prolonged LOS with a non-private insurance type.

## 4. Discussion

Lower back pain (LBP) is reported to be one of the most serious diseases affecting both health and function, in addition to being among the most expensive to treat [23]. There has been an increase in the use of various treatments over the past 20 years, as well as an increase in the cost of spinal care [6,24,25,26]. Despite these findings, studies demonstrate that the number of people suffering from disabling back pain has increased over the same period [26,27]. These developments are taking place in a context where health care is increasingly focused on providing value-based treatment to patients. Researchers, providers, and policymakers have an opportunity to design a uniform, value-driven, and digitalized spine care paradigm built on precision medicine. In the absence of studies pertaining to the length of stay, one of the most relevant patient-centered outcome measures, we investigated whether the prolonged length of stay could be predicted using artificial intelligence-based techniques. Our findings suggest that LOS can be predicted using data from lumbar decompression surgery patients. Although we only used a small number of cases, the algorithms showed satisfactory accuracies for the prediction task. As a result, our findings can serve as a foundation for larger multi-center prospective studies that collect data in order to develop more accurate models based on our findings.

Both the patient and the healthcare system can be significantly affected by a longer LOS following lumbar decompression surgery [28,29]. A recent study has shown that longer hospital stays are associated with higher complication rates and higher hospital costs, especially in neurosurgical and orthopedic spine cases. In particular, more prolonged LOS cases were more likely to be readmitted to the hospital. There is, however, the possibility that this may be caused by underlying comorbidities rather than LOS as such [30]. Further, there is evidence that prolonged hospital stay increases the risk of anemia requiring transfusion, impaired mental status, pneumonia, readmission, and hardware issues resulting in reoperation in patients who have undergone spine surgery [31]. One study of neurosurgical patients found that both physical therapy consultations and discharges to a specialist nursing facility were related to 2.4 days and 6.2 days longer length of stay, respectively [32].

Even though several risk factors have been identified for longer LOS following lumbar decompression surgery, no effective systematic model has been proposed to predict whether a patient will have a short or long LOS following this procedure. We only found one study applying machine learning techniques to predict prolonged LOS after lumbar spinal stenosis surgery [33]. Nevertheless, the authors reported an AUC of 0.54, which is insufficient, considering that an AUC of 0.5 translates to no classification capacity (not much better than a coin toss). In addition to identifying statistically significant clusters in our cohort, which support our hypothesis that prolonged hospital stays are associated with other features, the present study demonstrated that machine learning and deep learning techniques could be used to effectively predict short hospital stays versus long hospital stays. Along with contributing to a deeper understanding of a patient’s particular risk profile, these findings can assist in better resource allocation and discharge planning. The variables used in the machine learning method can be used to create an open-source web-based LOS prediction tool. Such a procedure would facilitate external validation of the provided models.

This study found that all algorithms in this study were able to predict and discriminate between classes with AUC values of 0.675–0.873. In this study, the findings compared favorably with those from other studies examining machine learning in orthopedic subspecialties such as spine surgery. Prior research utilizing machine learning has used tree-based methods or neural networks to predict intraoperative blood loss, prolonged hospital stay, patient-reported outcomes, and discharge dispositions in the field with similar or inferior results [15,34,35,36,37,38]. As the current study builds on previous work in this area, the results from the current study add to the growing body of evidence supporting the use of machine learning in orthopedic surgery. Also, according to Kobayashi et al., higher ASA class and longer operating time were associated with a prolonged LOS after spinal surgery, although our findings suggest that operation time contributes more significantly to the prolonged LOS [39]. According to Adogwa et al., surgeon practice style and preferences appear to be risk factors for prolonged LOS, which is consistent with our present finding that operating time is correlated with an increase in LOS [40]. It is important to acknowledge that operating time cannot be attributed solely to surgeon preference or style, as patient comorbidities may also contribute to longer surgical duration [41]. It is also important to consider the surgeon’s learning curves when assessing surgical prediction models. Overall, the operation time can be associated with multiple parameters which might also be of importance for LOS. The degree of invasiveness of spinal procedures is generally correlated with the outcome of the operation (blood loss, operation time, and complication rate). As such, the reduction of feature importance into a few parameters may be viewed as problematic as long as not all other potentially important features are included in an assessment, which is simply not feasible in a retrospective study design.

This risk assessment tool (RAPT) consists of six questions that have been validated for predicting outcomes of patients undergoing joint replacement surgery. In addition, it assists patients and physicians in identifying hurdles to discharge and can simplify the discharge process [42]. Likewise, the findings of this study could be incorporated into an equivalent tool. Quantifying the risks associated with surgery would be very helpful to both patients and physicians before surgery. In addition to more accurately setting patient expectations, this will allow the entire patient care team to develop an individualized care plan that is intended to reduce the length of stay while increasing patient safety and satisfaction.

Our results also indicate that preoperative CRP levels might be relevant for assessing the hospital length of stay in future prediction models. CRP is an acute-phase inflammatory cytokine that is released by the liver in response to interleukin-6 and other inflammatory factors [43]. It binds to phospholipids on bacterial surfaces, damaged or apoptotic eukaryotic cells, and nuclear debris in cells. When bound to Fc-gamma receptors, CRP activates the classical complement cascade and promotes phagocytosis [44]. CRP is a well-recognized marker of systemic inflammation. It is also used to predict cardiovascular events in nonsurgical populations based on different CRP risk cut-offs [45]. An increase in CRP levels may be caused by infection, inflammation, trauma, malignancy, or tissue infarction. In addition, an increase in CRP may be observed earlier in the course of a disease process than other non-specific markers (e.g., fever), and it may fall rapidly during the course of the recovery process [44]. CRP may be helpful as a screening test to detect inflammation early in the course of a disease process, as well as a monitoring tool to assess the effectiveness of treatment. There is evidence that CRP rises as a consequence of surgical trauma, and peaks 48 h after surgery [46,47]. In some patients, the CRP response may only be incomplete or may not occur at all [47]. Validated CRP risk categories were able to predict future cardiovascular events in apparently healthy individuals [48]. In previous studies of cardiac surgery patients, higher preoperative levels of CRP were associated with a greater risk of short- and long-term morbidity and mortality [49,50,51]. Further, a link has been reported between the peak postoperative CRP response and the degree of surgical trauma [52]. Although not all studies agree, minimal-invasive surgical procedures generally result in lower CRP levels than open procedures [53,54,55]. It appears that patients who have a high preoperative CRP are more likely to have a higher and later peak postoperative CRP than those who have a normal CRP [56]. The severity of inflammation seems to be not related to surgical operation grade only, suggesting that it may be due to both inflammatory co-morbidity and surgical trauma [57]. Overall, evidence regarding the impact of preoperative CRP in spinal surgery patients is limited. Although it might be intuitive that increased preoperative inflammatory states might affect the length of stay, this phenomenon needs to be validated in future prospective studies. In our algorithms, the inclusion of CRP as a laboratory marker led to an increase in diagnostic accuracy. Thus, it is recommended to include laboratory markers in addition to other data types (clinical data, imaging, genetics, histology) in future prediction models to evaluate whether the models’ diagnostic capabilities might improve. Further, this approach is helpful to determine the most impactful features in the dataset [15].

Despite the strength of the algorithms presented, the novelty of the approach, and the promising predictive results, the study does have some potential limitations. The algorithms must be based on a sample that is representative of the population of patients receiving spinal decompression surgery in order to be therapeutically beneficial. Because our data comes from a single, small study, its applicability to other institutions may be limited. As a result, external validation of the models given is required. Furthermore, data were collected retrospectively, lowering the evidence grade because the data may not be as reliable as data collected prospectively. We were also unable to increase the selection of variables to include other possibly important variables that could improve the model further due to the retrospective methodology. However, based on what we found in our prior work and a search of the literature [20], we set out to incorporate important variables.

## 5. Conclusions

Decompression of the lumbar spine is one of the most common procedures performed in spine surgery. The associated costs are highly dependent on the hospital length of stay. Patient-centered outcome prediction models to predict the length of stay can allow using the available societal healthcare resources effectively. This enables policymakers and providers to compare treatment strategies among different disciplines and to identify the relative priorities for optimal resource allocation among various interventions. Our results indicate that machine learning and deep learning techniques can effectively predict whether patients will have a prolonged length of stay. Implementing the provided algorithms into open-source software and external validation through large-scale prospective studies are warranted to introduce the provided prediction tools in clinics.

## Figures and Tables

**Table 1:** Descriptive statistics and pairwise comparisons of the clusters were obtained through the K-means cluster analysis, including the continuous scale feature hospital length of stay (LOS). **** *p* < 0.0001.

		BMI		Number_of_Levels		Op_Time		Preoperative_CRP		Age		Days_in_Hospital	
		Mean	Std. Deviation	Mean	Std. Deviation	Mean	Std. Deviation	Mean	Std. Deviation	Mean	Std. Deviation	Mean	Std. Deviation
Cluster	Low risk	27.39	5.670	1.04	0.242	55.04	29.086	4.45	13.325	57.2366	16.37208	11.70	5.482
	High risk	28.75	5.145	1.92	0.973	137.63	51.662	40.90	66.902	72.2849	11.97116	24.27	14.429
	Combined	27.74	5.562	1.27	0.656	76.13	51.034	13.76	38.912	61.0787	16.69396	14.91	10.247
	*p*-value	****	****	****	****	****	****	****	****	****	****	****	****

**Table 2:** Showing the descriptive statistics and pairwise comparison of the clusters obtained through the K-means cluster analysis, including the binary class feature of hospital length of stay. **** *p* < 0.0001.

		BMI		Number_of_Levels		Op_Time		Preoperative_CRP		Age		Frequencies	
		Mean	Std. Deviation	Mean	Std. Deviation	Mean	Std. Deviation	Mean	Std. Deviation	Mean	Std. Deviation	Normal	Prolonged
Cluster	High risk	28.31	5.649	1.62	1.022	104.92	58.253	23.72	51.250	73.2094	12.37884	0	63
	Low risk	27.53	5.531	1.14	0.382	65.58	43.776	10.11	32.706	56.6354	15.86603	172	0
	Combined	27.74	5.562	1.27	0.656	76.13	51.034	13.76	38.912	61.0787	16.69396	172	63
	*p*-value	****	****	****	****	****	****	****	****	****	****	****	

**Table 3:** Performance measures for the machine learning and deep learning algorithms to predict the length of hospital stay class (binary classification task). Analysis was done with k-fold cross-validation (k = 5). AUC: area under the curve; Accuracy: (TP + TN)/(TP + TN + FP + FN).

Algorithm	Accuracy	AUC
LogisticRegression	0.814	0.814
Random Forest classifier	0.808	0.826
SGD classifier	0.771	0.804
K-nearest neighbors	0.755	0.769
Decision Trees classifier	0.739	0.675
GaussianNB	0.755	0.799
SVM	0.771	0.794
Custom CNN	0.771	0.862

## Data Availability

The raw data are pseudonymized and available from the corresponding author on reasonable request. The python code and machine learning algorithm structures are available from: https://github.com/Freiburg-AI-Research (accessed on 16 May 2022).
